# The 14-item short health anxiety inventory (SHAI-14) used as a screening tool: appropriate interpretation and diagnostic accuracy of the Swedish version

**DOI:** 10.1186/s12888-022-04367-3

**Published:** 2022-11-14

**Authors:** Susanna Österman, Erland Axelsson, Nils Lindefors, Erik Hedman-Lagerlöf, Maria Hedman-Lagerlöf, Dorian Kern, Cecilia Svanborg, Volen Z. Ivanov

**Affiliations:** 1 Centre for Psychiatry Research, Department of Clinical Neuroscience, Karolinska Institutet, Tomtebodavägen 18A, SE-171 77 Stockholm, Sweden; 2 Division of psychology, Department of Clinical Neuroscience, Karolinska Institutet, Nobels väg 6, SE-17165 Stockholm, Sweden; 3 Division of Family Medicine and Primary Care, Department of Neurobiology, Care Sciences and Society, Karolinska Institutet, Alfred Nobels allé 23, SE-141 83 Huddinge, Sweden; 4 Liljeholmen Primary Health Care Clinic, Region Stockholm, Liljeholmstorget 7, SE-117 94 Stockholm, Sweden; 5 Gustavsberg Primary Health Care Clinic, Region Stockholm, Odelbergs väg 19, SE-134 40 Gustavsberg, Sweden; 6 Centre for Psychiatry Research, Department of Clinical Neuroscience, Karolinska Institutet, & Stockholm Health Care Services, Region Stockholm, Norra Stationsgatan 69, SE-113 64 Stockholm, Sweden

**Keywords:** Health anxiety, Hypochondriasis, Short health anxiety inventory, Psychometrics, Screening, ROC curve

## Abstract

**Background:**

The 14-item Short Health Anxiety Inventory (SHAI-14) is a common measure of health anxiety but its screening properties have not been studied. The aims of this study were to evaluate the SHAI-14 as a screening instrument, identify cut-offs for clinically significant health anxiety and investigate which scores correspond to different severity levels.

**Method:**

The study included 1729 psychiatric patients and 85 healthy controls. Participants completed the SHAI-14 and underwent a diagnostic interview. Cut-off scores were evaluated in three scenarios to approximate screening 1) in a psychiatric clinic, 2) in a low prevalence setting and, 3) of healthy volunteers (cut-off for remission). Receiver operating characteristics were used. Classification of severity was based on the distribution of SHAI-14 scores reported by patients with clinically significant health anxiety.

**Results:**

The area under the curve (AUC) values were high in all scenarios (above 0.95). The optimal cut-off scores on the SHAI-14 were 22 in the psychiatric context, 29 in a setting with low prevalence of psychiatric disorders and 18 versus healthy controls. SHAI-14 scores of 0–27 represented no or mild health anxiety, 28–32 moderate health anxiety and 33–42 substantial health anxiety.

**Conclusion:**

Brief self-report measures used as screening instruments are a simple way of gathering information about the presence of specific symptoms and thus a way to detect the likelihood of a diagnosis. The SHAI-14 shows evidence of good diagnostic utility in both clinical and non-clinical settings. However, which cut-off score is to be used, depends on the intended purpose and the setting where the cut-off is used.

**Supplementary Information:**

The online version contains supplementary material available at 10.1186/s12888-022-04367-3.

## Introduction

Health anxiety is characterized by a fear of or preoccupation with having or developing a severe disease. Health anxiety exists on a continuum, from minimal concerns or preoccupation with health, to pathological levels warranting a psychiatric diagnosis [1, 2], e.g. hypochondriasis (ICD-10 [3];) or illness anxiety disorder (DSM-5 [4];). Throughout this paper, we will refer to this pathological form as *clinically significant health anxiety.*

Clinically significant health anxiety is a relatively common condition with an estimated prevalence of 3.4% in the general population [5] and up to 20% in medical clinics [6]. Clinically significant health anxiety becomes chronic in approximately 50% of cases [7]. It leads to distress and functional impairment [5], is predictive of ischemic heart disease [8] and poor self-rated health [9], which in turn is a strong predictor of mortality [10]. Furthermore, due to high health care utilization among individuals experiencing clinically significant health anxiety, it is associated with high costs and strain on health care resources [11]. However, clinically significant health anxiety is responsive to treatment [12]. Therefore, identification of the condition is important from both an individual and a societal perspective.

Structured diagnostic interviews are helpful for assessing clinically significant health anxiety, but they are typically costly and time-intensive [13]. Brief dimensional self-report instruments are more easily administered in clinical and research settings, and can be used both to assess symptom severity and to determine the presence of clinically significant health anxiety. However, in order to identify a clinical diagnosis using a dimensional self-report instrument, a cut-off point which offers optimal discrimination between the presence and absence of a diagnosis needs to be determined.

One of the most widely used self-report measures for health anxiety is the 14-item Short Health Anxiety Inventory (SHAI-14 [14];). The SHAI-14 was designed to be a brief screening instrument able to differentiate between clinical and subclinical health anxiety independently of the individual’s physical health status, and has therefore been recommended for use in medical populations [15, 16]. The measure is also widely used in psychiatric [17–19] and non-clinical settings [20].

Although SHAI-14 has been shown to have good psychometric characteristics and is considered practical to use, it has not been evaluated as a screening tool [21]. A cut-off of 18 points for clinically significant health anxiety has been proposed [22], but we are not aware of any empirical basis for this specific cut-off. Further empirical investigation of cut-off points for clinically significant health anxiety on the SHAI-14 could be essential for screening purposes and for evaluating treatment effectiveness. There is also a need to investigate which scores on the SHAI-14 that correspond to mild, moderate and substantial levels of clinically significant health anxiety as these findings would significantly increase the scale’s clinical utility.

This study was conducted to determine optimal clinical cut-off points on the Swedish version of the SHAI-14. Our main research questions were:

1. What are the appropriate cut-off scores on the SHAI-14 for identifying clinically significant health anxiety?
2. When individuals with clinically significant health anxiety complete the SHAI − 14, what score is likely to correspond to mild, moderate, or substantial levels of clinically significant health anxiety, respectively?

## Material and methods

### Participants

#### Psychiatric sample

The first sample (*n* = 1729) in this study consisted of psychiatric patients who received internet-based treatment between April 2018–December 2020 at a psychiatric outpatient unit in Stockholm, Sweden (the Internet Psychiatry Clinic). This clinic offers self-referred patients internet-based cognitive behaviour treatment (ICBT) for multiple psychiatric disorders. The clinic’s inclusion criteria state that patients have to (a) have access to a computer with internet connection, (b) be 16 years of age or older, (c) not present with hindering psychiatric comorbidities (e.g., ongoing substance abuse, a psychotic syndrome and/or a moderate or high risk of suicide). All psychiatric patients in our sample had a primary diagnosis of clinically significant health anxiety, panic disorder (PD), social anxiety disorder (SAD) or major depressive disorder (MDD). The psychiatric sample was divided into (i) individuals with a primary or secondary diagnosis of clinically significant health anxiety (*n* = 471) and (ii) psychiatric patients without the diagnosis (*n* = 1258). Among individuals with clinically significant health anxiety (primary or secondary) the mean age was 35.4 (SD = 11.0; 66% female). The mean age of the psychiatric patients without clinically significant health anxiety was 33.9 (SD = 11.4; 61% female). For additional demographic and clinical characteristics, see Table 1.

**Table 1:** Participant characteristics

	Psychiatric patients with clinically significant health anxiety	Psychiatric patients without clinically significant health anxiety	Healthy volunteers
	*n* = 471	*n* = 1258	*n* = 85
Female, *n* (%)	311 (66%)	766 (61%)	49 (58%)
Age, *M* (*SD*), range	35.4 (11.0), 16–81	33.9 (11.4), 16–83	40.6 (12.0), 20–64
Married or de facto *n* (%)	391 (83%)	733 (58%)	64 (75%)
University studies or equivalent *n* (%)	345 (73%)	830 (66%)	68 (80%)
Employment *n* (%)			
Employed	374 (79%)	880 (70%)	69 (81%)
Student	72 (15%)	252 (20%)	6 (7%)
Other	25 (5%)	126 (10%)	10 (12%)
Psychotropic medication *n* (%)	292 (62%)	776 (62%)	0 (0%)
Diagnosis *n* (%)			
Clinically significant health anxiety	471 (100%)	0 (0%)	0 (0%)
Major depressive disorder	72 (15%)	631 (50%)	0 (0%)
Panic disorder	82 (17%)	317 (25%)	0 (0%)
Social anxiety disorder	37 (8%)	640 (51%)	0 (0%)
Generalized anxiety disorder	62 (13%)	114 (9%)	0 (0%)
Duration principal diagnosis *n* (%)			
< 1 year	48 (10%) ^a^	137 (11%)^b^	NA
1–5 years	109 (23%) ^a^	294 (24%) ^b^	NA
6–10 years	85 (18%) ^a^	178 (14%) ^b^	NA
> 10 years	225 (48%) ^a^	620 (50%) ^b^	NA
Symptom scales *M* (*SD*)			
SHAI-14	30.0 (5.5)	13.3 (7.6)	6.2 (3.5)
MADRS-S	17.3 (7.1)^c^	21.1 (8.2)	4,0 (3,6)
PDSS-SR	8.0 (5.4)^d^	5.9 (5.7) ^e^	0.2 (0.8)
LSAS-SR	27.4 (22.5)^f^	54.5 (30.1) ^g^	NA
WHODAS 2.0	18.1 (12.1) ^h^	26.1 (14.8)	NA
Severity rating *n* (%)			
CGI-S = 1	0 (0%)	0 (0%)	NA
CGI-S = 2	24 (5%) ^i^	29 (2%) ^j^	NA
CGI-S = 3	206 (45%) ^i^	398 (32%) ^j^	NA
CGI-S = 4	179 (39%) ^i^	611 (49%) ^j^	NA
CGI-S = 5	46 (10%) ^i^	183 (15%) ^j^	NA
CGI-S = 6	6 (1%) ^i^	21 (2%) ^j^	NA
CGI-S = 7	0 (0%)	0 (0%)	NA

*Note:* participants were coded as cases of clinically significant health anxiety if they had either a primary or secondary diagnosis of clinically significant health anxiety. Participants without clinically significant health anxiety had a primary diagnosis of PD, SAD or MMD

*Abbreviations*: *SHAI-14* Short Health Anxiety Inventory, *MADRS-S* The Montgomery-Åsberg Depression Rating Scale, *PDSS-SR* Panic Disorder Severity Scale, *LSAS-SR* Liebowitz Social Anxiety Scale, *WHODAS v 2* World Health Organization Disability Assessment Schedule, *CGI-S* Clinical Global Impressions-Severity Scale. *NA* Not applicable. Note that certain values are missing due to administrative changes at the clinic/difficulties extracting information

^a^*n* = 461

^b^*n* = 1229

^c^*n* = 463

^d^*n* = 447

^e^*n* = 996

^f^*n* = 462

^g^*n* = 1257

^h^*n* = 462

^i^*n* = 460

^j^*n* = 1242

#### Healthy volunteers

The second sample (*n* = 85), consisted of healthy volunteers who completed the SHAI-14 along with other psychiatric self-report questionnaires. They were recruited through advertisements in newspapers and social media. The recruitment was stratified by age and gender to ensure a similar distribution as the psychiatric sample. The mean age was 40.6 years (SD = 12.0; 58% female). For further demographic and clinical characteristics, see Table 1.

### Procedure

The study was approved by the Regional Ethics Review Board in Stockholm, Sweden (record number 2019–04295 and 2019–04194). All assessment interviews and self-report scales were administered in Swedish. Participants in the psychiatric sample completed a screening battery including the SHAI-14 and other psychiatric self-report questionnaires online about 3 weeks before a psychiatric assessment at the clinic (see 2.3.1 measures). Assessments were done by a psychiatrist, resident physician or psychologist, all under the supervision of a psychiatrist. Assessments included the complete Mini-International Neuropsychiatric Interview (M.I.N.I [23];) and an interview guide specifically targeting clinically significant health anxiety; the Structured Clinical Interview for the DSM-IV Axis I Disorders (SCID-I [24];) or the Health Preoccupation Diagnostic Interview (HPDI [13];). Thus all participants with clinically significant health anxiety had either a diagnosis of DSM-IV hypochondriasis, DSM-5 illness anxiety disorder or DSM-5 somatic symptom disorder (with health anxiety). Following the assessment, the severity of the primary disorder was rated by an assessor using the Clinical Global Impression Severity Scale (CGI-S [25];). All assessors had access to the results from the screening during assessment and had undergone training in how to administer the M.I.N.I. and how to assess the CGI-S.

The healthy volunteers in the study provided written consent and completed the SHAI-14 and other self-report questionnaires through the same web-based platform as used in the Internet Psychiatry Clinic. They thereafter underwent an assessment with a licensed psychologist in order to rule out the presence of psychiatric disorders. The assessments, made by three licenced psychologists, included the M.I.N.I [23] and the HPDI interview [13] and were conducted over the telephone which has been shown to be a valid method for conducting psychiatric diagnostic interviews [26]. All three psychologists had extensive experience in the assessment and treatment of psychiatric and somatoform disorders. Assessors were instructed not to look at the participants’ online scores on the self-report questionnaires prior to assessment.

A total of 100 potential participants were interviewed of which, 15 individuals were excluded due to the presence of psychiatric symptoms, leaving 85 participants who met inclusion criteria and were included as healthy controls in the study. The study participants received a movie ticket as compensation for taking part in the study.

### Measures

#### Self-report measures

##### Health anxiety

The Short health anxiety inventory (SHAI-14) is the abbreviated form of the original 64-item Health Anxiety Inventory (HAI). The instrument consists of 14 items measuring cognitive, affective and behavioural aspects of health anxiety, specifically assessing different aspects of health concerns during the past week [14]. It includes a 4-point scale, with items scored 0–3, with a scale range of 0–42. The SHAI-14 has been found to exhibit adequate internal consistency, (α = .81–.84) [16, 27] and good 1-week test-retest reliability *(r* = .87) [28]. Reliability estimates from the present study are described in the results section.

The Swedish SHAI-14 evaluated in this study is the shortened version of the Swedish 64-item HAI. The psychometric properties of the Swedish HAI have previously been investigated and the measure has demonstrated both good convergent and discriminant validity, good 2-week test-retest reliability (r = .81), as well as high internal consistency (α = .94–.95) [29].

##### Depressive symptoms

The Montgomery-Åsberg Depression Rating Scale – Self-report (MADRS-S [30];) is a 9-item self-report questionnaire which measures clinical characteristics of depression with a total score ranging 0–54,. The internal consistency of the MADRS-S in the present sample was adequate (α = 0.87).

##### Panic disorder symptoms

Panic Disorder Severity Scale (PDSS-SR) measures self-reported panic disorder severity [31]. It consists of 7 items, scoring 0–4, with a total score of 0–28. The internal consistency of the PDSS-SR in the present sample was good (α = 0.91). Social anxiety: Liebowitz Social Anxiety Scale (LSAS–SR), consists of 48 items measuring self-reported symptoms of social anxiety, with a global score ranging 0–144 [32]. The internal consistency of the LSAS-SR in the present sample was good (α = 0.97).

##### Disability

World Health Organization Disability Assessment Schedule 2.0 (WHODAS 2.0), is a measure that assesses disability and functional impairment [33]. We used the 12-item self-report version of the WHODAS, converting raw scores according to the recommended simple method into a summary score that ranges 0–100 (0 = no disability; 100 = full disability). The internal consistency of the WHODAS 12-item version in the present sample was satisfactory (α = 0.85).

##### Severity/global function

Clinical Global Impressions-Severity Scale (CGI-S) is a clinician-administered assessment of global functioning and severity of illness [25]. The CGI-S is a one-item measure evaluating the severity of psychopathology from 1 to 7 where the levels are named as *normal*, *not at all ill*, *borderline mentally ill*, *mildly ill*, *moderately ill*, *markedly ill*, *severely ill* and *among the most extremely ill patients*. The CGI-S has demonstrated reliability and validity for a range of psychiatric disorders [34–36].

#### Diagnostic assessment

The Mini International Neuropsychiatric Interview (M.I.N.I) is a structured interview used for assessing psychiatric disorders in accordance with the Diagnostic and Statistical Manual of Mental Disorders [23]. The MINI interview assesses the 17 most common psychiatric disorders and was administered to all participants in the study.

The Health preoccupation diagnostic interview (HPDI [13];) is an interview guide assessing SSD and IAD according to DSM-5, and has shown to have a high interrater reliability. The interview was used in order to establish clinically significant health anxiety among the psychiatric sample and to rule out significant health anxiety among the sample of healthy volunteers.

The Structured Clinical Interview for the DSM-IV Axis I Disorders (SCID-I) hypochondriasis module is a guide assessing hypochondriasis according to the DSM-IV [24]. The interview guide was used at the Internet Psychiatry Clinic for a short period of time (about 1.5 months) in order to establish clinically significant health anxiety.

### Statistical analyses

We conducted analyses in Stata 15.1, Jamovi 1.6.23.0, and R 4.1.0 with the lavaan package, in accordance with a pre-registered analysis plan (Open science framework: v3thp). First, we performed both a confirmatory factor analysis and an exploratory factor analysis to ensure that it was reasonable to represent the Swedish version of the SHAI-14 with one sum score. For a more detailed description of the factor analyses, see supplementary material. Internal consistency was reported as Cronbach’s alpha.

When established that it was reasonable to analyse the SHAI-14 as one sum score, we proceeded to the main analysis which pertained to the SHAI-14 as a screening tool, and the identification of cut-offs for clinically significant health anxiety. Since the properties of a diagnostic test depend on the composition of the individuals under study [37], we evaluated the SHAI-14 in the light of three scenarios. First, as a screening tool in the context of a psychiatric clinic where non-cases (i.e., individuals without clinically significant health anxiety) suffer from other psychiatric disorders. Second, as a screening tool in a context where, similar to the population at large, only a minority have substantial psychiatric symptoms, [38]. Third, we evaluated how well the SHAI-14 could discriminate individuals with clinically significant health anxiety from healthy individuals. See below for how these three scenarios were modelled statistically.

**Fig. 1:**
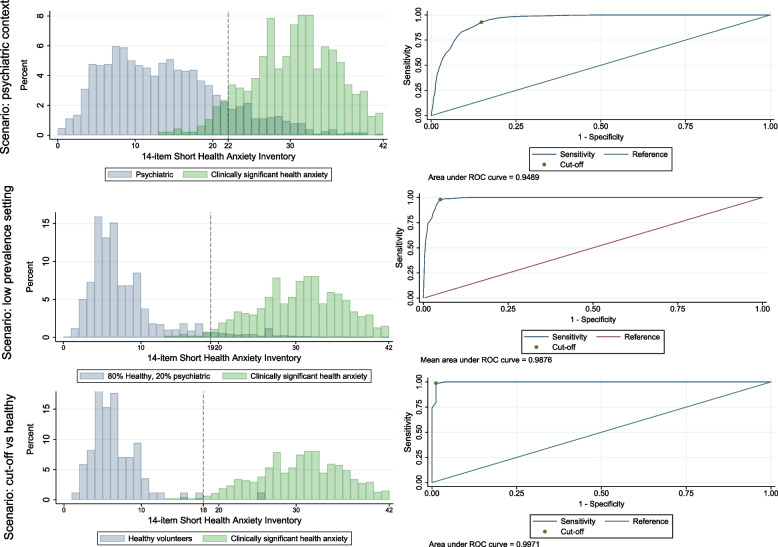
Receiver Operating Characteristic Curves and Distributions of Mean SHAI-14 Scores. *Note.* The figure demonstrates distribution of mean scores on the SHAI-14 in the three scenarios. The shaded part of the distributions shows the amount of overlap on mean scores on the SHAI-14 between the different samples. Clinically significant health anxiety refers to participants with either primary or secondary clinically significant health anxiety. ROC-curves for the whole range of cut-off points on the SHAI-14 in the three different scenarios are also presented and their respective AUC-values

Receiver Operating Characteristic (ROC) analyses were used and area under the curve (AUC) was calculated [39]. SHAI-14 score distributions and receiver operating characteristics are presented graphically (Fig. 1), and AUC estimates are presented with standard errors based on the DeLong et al. method [40]. Cut-offs to identify clinically significant health anxiety with a symmetrically weighted balance of sensitivity and specificity were selected using the Liu method [41], i.e., based on the largest sensitivity × specificity product. Confidence intervals for cut-offs, sensitivity and specificity were based on bias-corrected bootstrapping (2000 samples). In order to determine a cut-off to identify clinically significant health anxiety with as high sensitivity as possible, a cut-off value that maximizes sensitivity without reducing specificity below chance level was chosen.

Diagnostic accuracy of the SHAI-14 was investigated versus three reference groups: (i) psychiatric controls, (ii), a composite sample of healthy controls (80%) and randomly selected psychiatric controls (20%) and (iii) healthy controls. We based the second of these analyses on a resampling procedure where we drew 2000 samples with replacement, each consisting of 400 psychiatric patients with clinically significant health anxiety, 21 psychiatric patients without clinically significant health anxiety, and 84 healthy volunteers. Positive predictive values (PPVs) and negative predictive values (NPVs) were deduced algebraically based on sensitivity and specificity estimates. As the predictive value is related to disease prevalence, estimates of predictive values for a range of estimated prevalence rates were modelled. These were 1, 2 and 4%, which relates to the prevalence of clinically significant health anxiety in the general population [42], 20% in medical clinics [43] and 25% to psychiatry outpatient settings [44].

Last, in order to draft guidelines for the appropriate interpretation of SHAI-14 scores in terms of verbal labels for severity, we investigated the relationship between scores on the SHAI-14 and clinical ratings of the CGI-S. Using analogous methods as in the main analysis of clinical cut-offs (Liu, 2012), we estimated cut-offs on the SHAI-14 to differentiate between the categories: “mildly ill”, “moderately ill”, “markedly ill” and “severely ill” according to the CGI-S.

## Results

### Factor analysis and internal consistency

The confirmatory factor analysis showed that a one-factor model was not ideal according to the fit indices, see supplementary material. Therefore, we also explored the possibility of multidimensionality in an exploratory factor analysis. In summary, the SHAI-14 appeared to be 3-factorial but with a strong second-tier health anxiety factor which motivated further analysis of the SHAI-14 sum score for the purpose of this study. The internal consistency of the sum score (α = 0.96) and the potential subfactors (illness phobia: α = 0.90, worry: α = 0.92, bodily preoccupation: α = 0.88) were excellent.

### Diagnostic utility

The ROC curve, depicted in Fig. 1, represents graphically the performance of the SHAI-14 through the whole range of cut-off points in the three different scenarios. The AUC value was high in all scenarios; 0.95 (95% CI: 0.94, 0.96) in cases versus psychiatric non-cases, 0.99 (95% CI: 0.97, 1.00) in cases versus a mix of psychiatric and healthy non-cases and 1.00 (95% CI: 0.99, 1.00) in cases versus healthy volunteers.

### Scenario: psychiatric context

Versus other psychiatric patients, the symmetrically weighted clinical cut-off score was 22 (95% CI: 22, 25) with a sensitivity of 93% (95% CI: 86, 95%) and specificity of 85% (95% CI: 83, 90%). A cut-off of 13 points gave the maximum achievable sensitivity (100%) with a specificity above chance level (50.3%). In a psychiatric clinic with a true prevalence of clinically significant health anxiety of 20%, a cut-off of 22 on the SHAI-14 would yield a PPV of 61% and a NPV of 98%, correctly classifying 87% cases.

### Scenario: low-prevalence setting

We also estimated the clinical cut-off versus a combination of 20% psychiatric and 80% healthy controls. The idea here was to present a crude and tentative approximation of the kind of cut-off that would be suitable in a screening scenario where only a minority of non-cases have psychiatric symptoms, such in the occupational health setting or in the population at large where on average one in five adults experiences a mental disorder [38]. Here, the symmetrically weighted clinical cut-off was 19 (95% CI: 16–21), with a sensitivity of 98% (95% CI: 0.97–0.99) and specificity of 95% (95% CI: 0.90–0.98). Because PPVs were so low (see Table 2), especially with low prevalence rates, we also calculated a clinical cut-off that maximized specificity with a sensitivity above the level of chance. This clinical cut-off was 29 (95% CI: 25–31), with a sensitivity of 62% (95% CI: 0.57–0.67) and a specificity of 99% (95% CI: 0.97–1.00) which yielded higher PPVs, see Table 2.

**Table 2:** Summary Statistics for Different Cut-off Scores on the SHAI-14

Reference group	Cut-off type	Cut-off	Sensitivity	Specificity	Prevalence	PPV	NPV	Correctly classified
Psychiatric	Symmetrically weighted	22	0.93	0.85	0.20	0.61	0.98	0.87
Psychiatric	Symmetrically weighted	22	0.93	0.85	0.25	0.67	0.97	0.87
Psychiatric	High sensitivity	13	1.00	0.50	0.20	0.33	1.00	0.60
Psychiatric	High sensitivity	13	1.00	0.50	0.25	0.40	1.00	0.63
80% healthy, 20% psychiatric	Symmetrically weighted	19	0.98	0.95	0.01	0.17	1.00	0.95
80% healthy, 20% psychiatric	Symmetrically weighted	19	0.98	0.95	0.02	0.29	1.00	0.95
80% healthy, 20% psychiatric	Symmetrically weighted	19	0.98	0.95	0.04	0.45	1.00	0.95
80% healthy, 20% psychiatric	High specificity	29	0.62	0.99	0.01	0.36	1.00	0.99
80% healthy, 20% psychiatric	High specificity	29	0.62	0.99	0.02	0.53	0.99	0.98
80% healthy, 20% psychiatric	High specificity	29	0.62	0.99	0.04	0.72	0.98	0.98

*Note*. Symmetrically weighted cut-off type refers to the Liu-method. High sensitivity/specificity cut-off type refers to a cut-off value that maximize sensitivity/specificity without reducing sensitivity/specificity below chance level.

*Abbreviations*: *PPV* positive predictive value*, NPV* negative predictive value3.5 Scenario: clinical cut-off versus healthy controls

In a third scenario, we estimated the clinical cut-off versus healthy controls because this may be seen as a credible cut-off for remission after treatment. This symmetrically weighted cut-off was 18 (95% CI: 18, 18) with a sensitivity of 99% (95% CI: 97, 100%) and specificity of 99% (95% CI: 96, 100%).

### Classification of severity

As detailed in the introduction, a limitation of the literature is the scarcity of guidelines for interpreting SHAI-14 scores in terms of severity. We related self-report scores on the SHAI-14 to clinicians’ severity ratings on the CGI-S finding that a SHAI-14 score of 30 points was ideal for differentiating between “mild” and “moderately ill”, a score of 33 points was ideal for differentiating between moderately and “markedly ill”, and that a score of 34 points was ideal for differentiating between markedly and “severely ill”. In this study, the mean SHAI-14 score among treatment-seeking psychiatric patients with clinically significant health anxiety was 30.5, which is also roughly the mean level of health anxiety that has been reported in most clinical trials for clinically significant health anxiety [17–19, 45]. This would imply that roughly half of those treated for clinically significant health anxiety in specialized settings should be classified as having mild symptoms, which we did not find reasonable. In a non-planned analysis, we therefore simply divided the distribution of SHAI-14 scores reported by patients with principal clinically significant health anxiety into three equally sized strata based on quantiles, 33%/33%/33%, so as to base the classification on this. Here, we found that the 33rd-34th percentile corresponded to a SHAI-14 score of 28 and that the 67th–68th percentile corresponded to a score of 33. Tentatively then, a more reasonable lower bound for moderate as opposed to mild health anxiety could be 28 points, which means that the individual is likely to not belong to the third with mildest symptoms in the population of persons with clinically significant health anxiety. A score of 33 or higher would indicate “markedly ill” on the CGI-S and also mean that the individual is likely to belong to the highest third of a psychiatric clinically significant health anxiety sample.

## Discussion

The purpose of this study was to examine the utility of the SHAI-14 as a diagnostic instrument, and to determine optimal clinical cut-off scores. This was investigated within the framework of three scenarios, because optimal cut-offs and measures of efficiency such as sensitivity and specificity depend on the composition of the population where the cut-off is used [37, 46]. All AUC values were above 0.95 indicating that the SHAI-14 appears to be suitable as a screening tool. In the psychiatric setting, the optimal symmetrically weighted clinical cut-off was found to be 22. That is, patients with a sum score of 22 or higher are likely to have clinically significant health anxiety whereas those who score below this cut-off are very unlikely to have it. In a scenario similar to an occupational health setting or in the general population, where most individuals are healthy but around 20% exhibit psychiatric problems, an optimal symmetrically weighted clinical cut-off would be lower, probably around 19, and a cut-off resulting in very high specificity would probably have to be as high as 29. The optimal, symmetrically weighted cut-off versus healthy controls was 18. This means that individuals who score 17 or lower on the SHAI-14 are unlikely to suffer from clinically significant health anxiety. While acknowledging that it is possible to score high on a health anxiety scale for other reasons than health anxiety, our best approximation is that SHAI-14 scores of 0–27 typically represent no or mild health anxiety, scores of 28–32 typically represent moderate health anxiety and scores of 33–42 typically represent substantial health anxiety. Overall, this study illustrates that the SHAI-14 can be used to identify cases of clinically significant health anxiety, and that the optimal cut-off depends on its intended purpose and the setting where the cut-off is used.

### Factor structure of the SHAI-14

To our knowledge, this is the first study investigating the factor structure of the SHAI-14 in a pooled sample of psychiatric patients and healthy individuals. Our results showed the SHAI-14 to have one overarching second-tier health anxiety factor, and three highly correlated subfactors: Illness phobia, Worry and Bodily preoccupation. This is in contrast to the original publication, where the SHAI-14 was developed with the aim of being unifactorial [14], which has also been replicated in one study [47]. Other authors have suggested the SHAI-14 to consist of two factors [15, 48]; one factor assessing the tendency to experience unwanted health-related thoughts (i.e., Thought Intrusion) and one referring to the fear of having a serious medical condition (i.e., Fear of Illness). In these studies, the SHAI-14 was administered to samples with different medical conditions and healthy individuals. The inconsistent result may imply that the SHAI-14 items load into different factors when administered to a psychiatric sample as opposed to other groups. Also, the divergent factor structure could also be affected by potential linguistic differences between the Swedish SHAI-14 and the original instrument. Finally, we found that the internal consistency of the full scale and the subfactors was excellent. In summary, although our results suggested a three-factor solution, the strong second-tier health anxiety factor clearly supports scoring the SHAI-14 as a total score between 0 and 42.

### Clinical cut-off in the psychiatric setting

ROC-curves showed that the SHAI-14 is well suited for screening for clinically significant health anxiety in the psychiatric setting, with an optimal, symmetrically weighted cut-off score of 22 points combining high sensitivity (93%) and high specificity (85%). As a comparison, in a study of the slightly longer 18-item SHAI, where the control group also consisted of individuals with psychiatric disorders, a cut-off score of 27 yielded a sensitivity of 85.7% and a specificity of 77.9% [49]. Our slightly higher values ​​may be explained by the fact that our control group comprised a mix of patients with anxiety and depression as opposed to only including patients with anxiety disorders.

Another aspect of a test’s diagnostic utility is the PPV and NPV. However, it is important to evaluate the predictive values of a test based on the true prevalence of the disorder in the specific context where it will be used [50]. For example, in order to calculate the proportion of those scoring above the cut-off who actually have clinically significant health anxiety (i.e., the PPV), it is necessary to assume that a certain proportion of those screened are true cases with clinically significant health anxiety. In a psychiatric clinic, we believe that it can be reasonable to expect a true prevalence of 20%, which would mean that a cut-off score of 22 points on the SHAI-14 yields a PPV of 61% and an NPV of 98% (see Table 2). That is, if the true prevalence of clinically significant health anxiety in the clinic is 20%, of those scoring at least 22 points on the SHAI-14, 6 out of 10 could be expected to be actual cases with clinically significant health anxiety as opposed to for example cases with panic disorder. Moreover, nearly all patients with a score below 22 could be expected not to be clinically significant health anxiety cases. In summary, our results indicate that the SHAI-14 has considerable utility as a screening instrument for clinically significant health anxiety in clinical settings when a cut-off point of 22 is used.

Depending on the specific need in the clinical or research context, a cut-off point that maximizes sensitivity is sometimes preferred, even if that means a limited specificity [51]. This is often the case when a scale is used for screening purposes and if the test is inexpensive and simple. Based on our analyses, if high sensitivity would be the most valued aspect, a cut-off score of 13 on the SHAI-14 is proposed, correctly classifying 60–63% (depending on the prevalence rate), yielding a NPV as high as 1.0.

### Tentative investigation of cut-offs for low-prevalence settings

This study focused on screening in a psychiatric setting but in a secondary analysis we also made an attempt to preliminarily estimate what an appropriate cut-off could be when using the SHAI-14 in a screening scenario where only a minority of non-cases have psychiatric symptoms. This was done through simulations where the contrast group included 80% healthy volunteers and 20% psychiatric patients without clinically significant health anxiety. Preliminarily, our results showed that a cut-off of 19 points would be appropriate if sensitivity and specificity are weighted equally. However, for epidemiological purposes a cut-off that maximizes specificity could be of higher value in order to avoid many possibly false positive cases. In order to reach a more acceptable specificity, a cut-off score of 29 points would be needed, where 97–99% would be correctly classified (depending on the modelled prevalence rates).

### Clinical cut-off versus healthy controls: proposed criterion for remission

In treatment research, self-report instruments are often used to estimate whether a psychiatric diagnosis is still present after treatment. For this purpose, we investigated what score on the SHAI-14 that best discriminated between individuals with clinically significant health anxiety and healthy individuals. A symmetrically weighted cut-off point of 18 with a sensitivity and specificity of 99% would be ideal in this context and the same cut-off score has previously been proposed to identify individuals meeting DSM-IV criteria for hypochondriasis [22]. In sum, 18 points on the SHAI is probably the best threshold if one is interested in using a cut-off in research in order to establish whether an individual has achieved diagnostic remission.

### Interpretation of the SHAI-14 in terms of severity

Finally, we made an attempt to classify the level of severity according to SHAI-14 scores. Our results indicated that SHAI-14 scores of 0–27 typically represent no or mild levels of health anxiety, scores of 28–32 typically represent moderate health anxiety, and scores of 33–42 typically represent substantial health anxiety. As pointed out above, in our first analyses, we were surprised to find 30 to be the optimal cut-off to discriminate “mild” from “moderate” illness according to CGI-S, as it did not seem reasonable for us, considering that 30 points appears to approximate the average symptom level seen in clinical trials and specialist psychiatric clinics. As this was conducted in a psychiatric setting with psychiatrists with experience of patient groups typically having more debilitating symptoms, such as persons with psychotic or bipolar disorders, one tentative explanation of the finding is that in this broader psychiatric context, a substantial proportion of health anxiety cases might be regarded as having mild symptoms. Our proposal for interpretation of the SHAI-14 in terms of severity should be interpreted with caution. In order to further evaluate these empirically, it would be important to validate them against a clinician-administered severity rating instrument specifically targeting health anxiety such as the hypochondriasis version of the Yale-Brown Obsessive-Compulsive Scale (H-YBOCS) [52, 53] and use existing benchmarks on the instrument for defining clinical severity [54].

### Clinical implications

Although relatively common, clinically significant health anxiety often goes undiagnosed in clinical settings [55]. Screening for clinically health anxiety is important as it is a persistent condition, leads to both personal suffering and impairment and places a significant burden on health care services [5, 55]. Brief self-report measures used as screening instruments are a simple way for gathering information about the presence of specific symptoms and thus a way to detect the likelihood of a diagnosis. This information can then form the basis of a decision as to whether a more thorough diagnostic assessment should be carried out. In the present study, three different cut-off scores have been calculated. Which one should be used depends on the context in which the instrument is used:

First, clinicians working at a psychiatric clinic could use the SHAI-14 as a part of a screening battery, where a cut-off score of 22 and above is a clear indicator that the individual who completed the instrument has clinically significant levels of health anxiety. In the next step the clinician can decide on whether to go through with a diagnostic interview.

Second, if the SHAI-14 is used in a context such as the population at large as a way to estimate prevalence of the condition, where no clinical interview would be conducted as the next step, a cut-off score of 29 should be used instead in order to estimate clinically significant health anxiety.

Third, if he SHAI-14 is used as a way to evaluate treatment effect, a score under 18 on the instrument is probably the best cut-off to use in order to estimate diagnostic remission.

Finally, one should always have in mind the limitations inherent in the use of self-reporting instrument such as the dependence of cooperation and reading ability of the person answering them. Furthermore, because brief self-report scales do not include the whole spectrum of symptoms of the target condition, they need to be followed up with a more thorough assessment so that a diagnosis can be made.

### Strengths and limitations

This study had notable limitations. Data was collected by clinicians who had undergone training, were supported by check-lists and supervision, but no formal interrater-reliability tests concerning diagnoses were done. As pointed out in the methods, in this study, clinically significant health anxiety could mean that the patient met criteria for a clinical diagnosis of DSM-IV hypochondriasis, IAD or SSD according to DSM-5. Also, the sample consisted of patients seeking treatment which could limit the generalizability of the results to individuals with clinically significant health anxiety in general such as outside a psychiatric context or in a medical setting. Nevertheless, a strength with the present study is that all participants underwent a thorough clinical assessment with a clinician based on a structured interview, which is the gold standard of diagnostic interviewing in psychiatric research [56] and we believe that the assessment of these disorders was valid and reliable enough not to jeopardize our results on the whole. Concerning further criteria for accuracy of diagnostic studies, the time period between the administration of the SHAI-14 to the reference test was deemed sufficiently short to ensure that the condition did not change during the period [57]. Additionally, the analyses were made using a relatively large sample and our control group consisted of both individuals with other psychiatric disorders and of healthy volunteers.

Another obvious limitation of this study is that our investigation into cut-offs for the low-prevalence scenario was based entirely on a highly speculative simulation, which means that all estimates derived from this part of the study should be regarded as tentative. However, investigating the SHAI-14 in different simulated scenarios was an attempt to show how optimal cut-offs ​​can vary depending on the context in which the instrument is used. Future studies should evaluate the SHAI-14 in larger, randomly selected samples from the population in order to further explore the psychometric properties of the SHAI-14. Another task for further research would also be to investigate clinical cut-offs in a sample of individuals with medical disorders.

## Conclusions

This study showed evidence for the diagnostic utility of the SHAI-14 in the assessment of clinically significant health anxiety. Based on the results, we conclude that the SHAI-14 is a measure with sound psychometric properties that can be used as a screening instrument in both clinical and potentially non-clinical settings to identify individuals with clinically significant health anxiety.

## Supplementary Information

**Additional file 1.**

## Data Availability

The data that support the findings of this study are available from Karolinska Institutet but restrictions apply to the availability of these data, which were used under license for the current study, and so are not publicly available. Data are however available from the authors upon reasonable request and with permission of Karolinska Institutet.

## Abbreviations

SHAI-14: The 14-item Short Health Anxiety Inventory
AUC: The area under the curve
CFI: Comparative fit index
CGI-S: Clinical Global Impressions-Severity Scale
HAI: Health Anxiety Inventory
HPDI: The Health Preoccupation Diagnostic Interview
IAD: Illness anxiety disorder
ICBT: Internet-based cognitive behaviour treatment
LSAS-SR: Liebowitz Social Anxiety Scale
MADRS-S: The Montgomery-Åsberg Depression Rating Scale
MDD: Major depressive disorder MDD
M.I.N.I: Mini-International Neuropsychiatric Interview
NPV: Negative predictive values
PD: Panic disorder
PDSS-SR: Panic Disorder Severity Scale
PPV: Positive predictive values
RMSEA: Root mean square error of approximation
ROC: Receiver Operating Characteristic
SAD: Social anxiety disorder
SCID-I: Structured Clinical Interview for the DSM-IV Axis I Disorders
SRMR: Standardized root mean square residual
SSD: Somatic symptom disorder
TLI: Tucker–Lewis index
WHODAS v 2: World Health Organization Disability Assessment Schedule
