# Supplementary material for: The 14-item short health anxiety inventory (SHAI-14) used as a screening tool: appropriate interpretation and diagnostic accuracy of the Swedish version
Source: BMC Psychiatry. 2022 Nov 14;22:701. doi: 10.1186/s12888-022-04367-3 (PMC9664720; doi:10.1186/s12888-022-04367-3)
Supplement: Supplementary file 1 — Additional file 1. [file 12888_2022_4367_MOESM1_ESM.docx]

Supplemental Information for The 14-item Short Health Anxiety Inventory (SHAI) Used as a Screening Tool: Appropriate Interpretation and Diagnostic Accuracy of the Swedish version

**Material and methods**

Statistical analyses

A confirmatory factor analysis was conducted, using weighted least squares means and variance adjusted estimation (1). Model fit was assessed in terms of the Comparative fit index (CFI), Tucker-Lewis index (TLI), Standardized root mean squared residual (SRMR), and Root mean square error of approximation (RMSEA) where common rules of thumb for adequate fit are a CFI≥0.90 or ideally ≥0.95, a TLI≥0.90 or ideally ≥0.95, an SRMR ≤.08, and an RMSEA ≤.08 or ideally ≤ .06 (2, 3). Because model fit was not ideal, an exploratory factor analysis was also conducted based on principal axis factoring with promax rotation with the number of factors chosen based on the inflection point of the scree plot, and also based on parallel analysis which is one of the most accurate methods for determining the number of factors to retain (4). This was done in a composite sample of the 70 % of the total sample (selected consecutively based on date of screening), stratified over primary diagnosis, and was then validated using confirmatory factor analysis in the remaining 30 %.

**Results**

Factor analysis

Upon visual inspection, items 8, 9, and 12 showed signs of skewness. In multivariate normality checks, Mardia's test, Henze-Zirkler's test, and Royston's test were also indicative of non-normality. We therefore conducted confirmatory factor analyses using weighted least squares means and variance adjusted estimation (1). Confirmatory factor analysis was indicative of the one factorial model having borderline adequate fit (χ²[77] = 1724, p < .001; CFI = 0.99; TLI = 0.98; SRMR = 0.035; RMSEA = 0.109, 90% CI [0.104, 0.113]) with factor loadings of 0.70 – 0.94. Based on modification indices we added covariance between items 10 and 13 (possibly bodily preoccupation for other reasons than health anxiety, for example panic disorder) and items 1 and 7 (possibly general propensity for worry) resulting in slightly improved and borderline adequate fit (χ²[75] = 1246, p < .001; CFI = 0.99; TLI = 0.99; SRMR = 0.032; RMSEA = 0.093, 90% CI [0.088, 0.097]). Because the fit of the one factor model was not ideal, we also explored the possibility of multidimensionality in exploratory factor analysis (KMO=0.97). Based on the knee of the scree plot (see Figure 1), the SHAI-14 still appeared to be clearly unifactorial. However, based on parallel analysis, three Eigenvalues were stronger than those of the simulated data. The resulting 3-factor solution could be interpreted as illness phobia (item 5,9,11,12 ≥0.40, 6,8,14≥0.30), worry (item 1,4,6,7 ≥0.40, 2,5,8 ≥ 0.30), and bodily preoccupation (item 3,10,13,14 ≥ 0.40, 2 ≥ 0.30). In the validation data, model fit was adequate (χ²[74] = 311, p < .001; CFI = 0.99; TLI = 0.99; SRMR = 0.029; RMSEA = 0.077, 90% CI [0.068, 0.086]) and the interfactor correlation was high (*r* = .91-.95) with all items having high loadings on a second-tier health anxiety factor if this was added (0.94-0.98).

**Figure 1**

*Scree Plot of the SHAI-14* *Exploratory Factor Analysis*


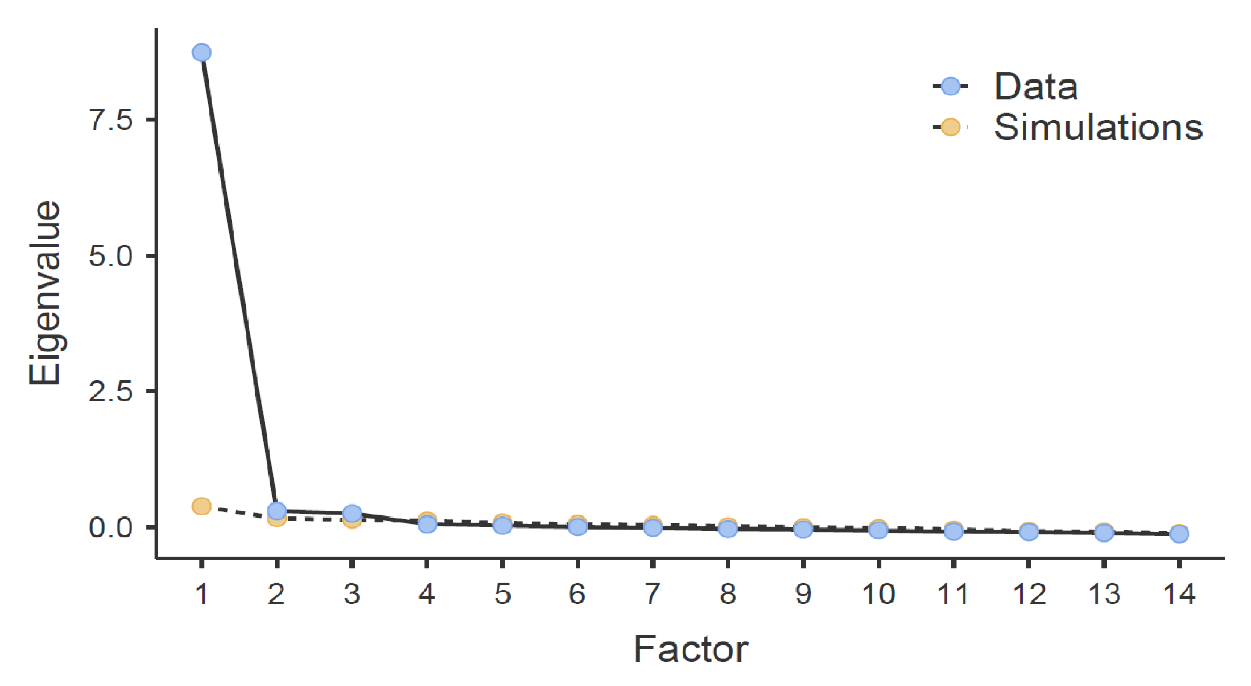


*Note.* Axis X represents number of possible factors. The scree plot indicates a 1-factor solution.

**References**

1. Li CH. Confirmatory factor analysis with ordinal data: Comparing robust maximum

likelihood and diagonally weighted least squares. Behav Res Methods. 2016;48(3):936-49.

2. Hu Lt, Bentler PM. Cutoff criteria for fit indexes in covariance structure analysis:

Conventional criteria versus new alternatives. Structural equation modeling: a

multidisciplinary journal. 1999;6(1):1-55.

3. Marsh HW, Hau K-T, Wen Z. In search of golden rules: Comment on hypothesis-

testing approaches to setting cutoff values for fit indexes and dangers in overgeneralizing Hu

and Bentler's (1999) findings. Structural equation modeling. 2004;11(3):320-41.

4. Zwick WR, Velicer WF. Comparison of five rules for determining the number of

components to retain. Psychological bulletin. 1986;99(3):432.
